# On the Physiology of the Iris

**Published:** 1828-04

**Authors:** Egerton A. Jennings

**Affiliations:** Member of the Royal College of Surgeons.


### THE LONDON
### Medical and Physical Journal.

No. 350, VOL. LIX.] APRIL, 1828. [No. 22, *New Series.*

For many fortunate discoveries in medicine, and for the detection of numerous errors, the world is indebted to the rapid circulation of Monthly Journals; and there never existed any work, to which the Faculty, in Europe and America, were under deeper obligations, than to the Medical and Physical Journal of London, now forming a long, but an invaluable, series.—RUSH.

### ORIGINAL PAPERS,

AND

### CASES OBTAINED FROM PUBLIC INSTITUTIONS AND OTHER AUTHENTIC SOURCES.

### IRIS.

*On the Physiology of the Iris.* By EGERTON A. JENNINGS, Member of the Royal College of Surgeons.

PHYSIOLOGISTS, in their attempts to account for the motions of the iris which produce contraction and dilatation of the pupil, have generally adopted one of the two following theories: either they have supposed the iris to be composed of two sets of muscles, one arranged circularly, whose contractions diminish the aperture of the pupil; the other composed of straight fibres, whose contractions dilate the pupil; or else they have supposed the substance of the iris to be an erectile tissue, its dilatation or contraction depending on the quantity of blood circulating through its vessels.

There are, however, numerous facts that cannot be accounted for by either of these theories. Thus, if the former theory be true, the dilatation of the pupil caused by a large dose of digitalis, or by the local application of belladonna, and the same effect as caused by pressure on the brain and by death, can only be accounted for by supposing its muscles to be deprived of nervous influence. That being the case, the same cause that deprived the sphincter muscle of its nervous influence should also deprive the straight fibres of theirs, and the iris should remain in an inactive state, between contraction and dilatation; or it should become paralysed, in whatever state it might be at the time the cause of the paralysis was applied. But this we know is not the case, —the pupil is always dilated.

Again, when the anterior part of the iris is irritated, contraction of the pupil takes place. If there were both straight and circular muscular fibres, both being equally stimulated, the iris should remain stationary, or alternately dilate and contract, from the two muscles alternately acting, as the flexor and extensor muscles of a limb do, when the nerves leading to them are stimulated. Supposing the sphincter muscle to be much the stronger of the two, the contraction might arise from its overpowering the straight fibres; but then, when belladonna is applied to the eye, the straight muscle, being the weaker, should be first paralysed, and we should have the pupil more contracted, at least for some time before the dilatation commenced. A theory which cannot account for such important facts as these, cannot be correct. Let us now examine the other, which supposes the movements of the iris to depend upon the injection of its vessels.

The first objection to this theory is, that we have no proof of an increased supply of blood being sent to the part at the time of its dilatation; while, in the next place, we have distinct proof that an increased quantity of blood may be circulating through the iris, and yet the pupil be preternaturally dilated. This is the case when belladonna is applied during inflammation of the iris. Here, though we see the anterior ciliary arteries injected with red blood, yet the pupil is much dilated. This it most certainly should not be, if the dilatation of the iris depended on the quantity of blood circulating through its vessels.

It may be asked, can any theory be offered capable of accounting for these facts? I think there may; and one that in a great measure removes the perplexity that has surrounded this interesting subject. But, before offering any theory, let us examine the anatomical structure of the parts in question, and endeavour from that to draw some legitimate conclusion concerning the function of the different parts.

If we examine the anterior surface of the iris, we can plainly distinguish a small portion, extending round the pupillary margin, differing in colour and appearance from the other part. This, in the best delineations of the iris, is represented as composed of circular fibres, and I am inclined, for reasons I shall hereafter assign, to believe it to be a circular muscle.* The nerves distributed to the iris arise from

* By the term muscle, I would be understood to mean an arrangement of animal fibres having a power of contractility dependent on nervous influence two sources: from the third pair, and from the first division of the fifth pair of nerves. The latter, as far as we are acquainted with its functions, as distributed to the upper part of the face and head, is entirely a nerve of sensation, while the former only presides over muscular motion.

The distribution of a muscular nerve to this organ would, of itself, be a strong presumptive evidence that some part possessing muscular power did exist, and would render it probable that the motions of the iris depended in some way on muscular action. This supposition is rendered still more probable by the fact that, in some animals, as the rabbit and guinea-pig, the third is the only nerve that sends filaments to the iris. Hence, as contraction and dilatation of the iris takes place in them, its simple motions must be radically independent of the fifth pair of nerves, however much they may influence the time and circumstances under which it acts.

Having given the reasons that principally induced me to think the motions of the iris dependent on muscular action, I shall next endeavour to point out the mode in which its different motions are effected.

The dilatation of the iris, and consequent contraction of the pupil, I believe entirely to depend upon the contraction of a sphincter muscle, situated at the pupillary edge of the iris. This I am induced to believe from the fact that all stimuli applied to the nerves of the part occasion this contraction; and any thing that tends to paralyse these nerves, as the application of belladonna, pressure on the brain, and death, all deprive this muscle of the power of action. On the other hand, I believe the contraction of the iris, and consequent dilatation of the pupil, to depend on the elasticity of the parts situated between the sphincter muscle and ciliary margin, a power distinct from muscular contraction, as it is independent of nervous influence, and even of life itself. This would account for the dilatation of the pupil when the supply of nervous influence is cut off; and it is not the only case in which nature has placed simple elasticity as an opponent to muscular contraction. In respiration, the elasticity of the cartilages of the ribs draws down the chest when the muscles of inspiration relax; and, from their elasticity continuing after the inspirators are deprived of all supply of nervous energy, expiration is the last act of life. The contraction of the iris taking place at, and continuing after, death, certainly shows that it also depends on elasticity, a power uninfluenced by the vitality of the parts.

I had recently a case under my care, showing very decidedly the influence of the ciliary nerves on the contraction of the iris. The following I extract from my notes of the case:

"J. Green, a labourer, was accidentally struck on the upper part of the eye by the lash of a carter's whip. I saw him soon after the accident, and found the upper eyelid cut. On the upper surface of the globe of the eye, about two lines from the cornea, there was a slight extravasation of blood on the sclerotica, beneath the conjunctiva. The iris presented a curious appearance: it was contracted along its upper margin, while the rest of it was dilated; the pupil was thus drawn nearly to the ciliary margin at the upper part of the eye, to an extent corresponding with the extravasation on the sclerotica. On exposing the eye to a strong light, the upper portion of the iris was fixed, though the other parts dilated and contracted as usual."

From the paralysis of the portion of the iris being so immediate, and so exactly corresponding with the extent of injury done to the sclerotica, I think myself justified in concluding that it must have been caused by concussion of the ciliary nerves; and the iris being so closely contracted at the part corresponding to the injury, shows that its contraction is different from muscular action, being independent of nervous influence.

Mr. Lawrence has met with two cases, showing still more decidedly that the dilatation of the iris depends on muscular action, (at least on a power which, like it, owes its action to the influence of nerves of muscular motion:) he does not, however, seem to have drawn any conclusion from them. He says, "I have seen two cases* in which there was a paralysis of all the parts supplied by the nerves of the third pair, viz. three of the recti muscles, one of the oblique muscles, and the levator palpebra superioris; so that the upper eyelid could not be elevated, and the globe was drawn outwards by the external straight muscle. In both instances the pupil was largely dilated. You may, perhaps, suspect that the optic nerve may have been insensible; but this was not the case: for, in one of those instances, when the patient looked through a minute opening in a card, producing what may be called an artificial contracted pupil, vision was perfect."

These cases are, in some measure, similar to the one I met with, as far as the iris is concerned; but they are much more satisfactory, as the paralysis was more complete; and, as the fifth pair of nerves appear to have been in a perfect state, they

* Lancet, vol. ix. p. 245.

prevent the possibility of our attributing the contraction of the pupil to their influence.

The fact that the pupil is contracted during sleep may be thought opposed to the idea that its contraction is dependent on the action of a sphincter muscle; for, at that time, muscular energy seems to be suspended. I do not, however, apprehend it to be any objection. The sphincter muscles differ from the straight muscles, inasmuch as they never appear to be fatigued by action. Thus, when the general muscular system is in a state of rest during sleep, the sphincter ani and the sphincter vesicæ both continue to act: so does also what I have termed the sphincter muscle of the iris. This, therefore, seems only to render it more closely allied to the other sphincter muscles.

What then, it will be asked, is the function performed by the branches of the fifth pair of nerves distributed to the iris? That they are sent there for an important purpose, no one can doubt. I have observed some facts which may, I trust, tend to throw some light on this interesting subject. But this paper is already too long to permit of my entering upon that subject at the present time. I shall, however, continue my investigations, and probably, at some future period, communicate the result of my observations.

*Leamington Spa; January 5th,* 1828.

